# Exploratory Investigation Into Perioperative Treatment Strategies for Potentially Resectable Stage III–N2 Driver Gene–Negative Non–Small Cell Lung Cancer in the Immunotherapy Era

**DOI:** 10.1002/cam4.71696

**Published:** 2026-03-08

**Authors:** Jiarui Zhao, Baiyang Huang, Min Li, Xingpeng Wang, Jingyu Zhu, Kaiyue Wang, Jing Xu, Xiaohan Wang, Menglin Bai, Guoxin Cai

**Affiliations:** ^1^ Department of Radiation Oncology Shandong Cancer Hospital and Institute, Shandong First Medical University, and Shandong Academy of Medical Sciences Jinan Shandong China; ^2^ School of Clinical Medicine Shandong Second Medical University Weifang China; ^3^ Shandong University Cancer Center Cheeloo College of Medicine, Shandong University Jinan Shandong China; ^4^ Department of Radiation Oncology Qilu Hospital of Shandong University Jinan Shandong China

**Keywords:** definitive chemoradiotherapy, metastatic N2 lymph node, neoadjuvant chemoimmunotherapy, resectable non–small cell lung cancer

## Abstract

**Background:**

Comparisons among treatment strategies for potentially resectable Stage III–N2 driver gene–negative non–small cell lung cancer (NSCLC) remain limited. We evaluated three treatment strategies—neoadjuvant chemoimmunotherapy followed by surgery (NCIT+surgery), NCIT followed by chemoradiotherapy (NCIT+CRT), and definitive concurrent chemoradiotherapy followed by immunotherapy (CRT + IT)—to investigate whether CRT could represent a feasible option to surgery.

**Methods:**

Patients with Stage III–N2 NSCLC from two institutions (2019–2024) were retrospectively enrolled. Primary endpoints were progression‐free survival (PFS) and overall survival (OS). Subgroup analyses included metastatic N2 lymph node status, stage, programmed death ligand 1 expression, radiographic response to NCIT, and adjuvant or consolidation immunotherapy after surgery or CRT. Baseline differences were balanced 1:1 by propensity score matching (PSM). Survival was estimated by the Kaplan–Meier method and compared via the log‐rank test.

**Results:**

A total of 363 patients were included: NCIT+Surgery (*n* = 176), NCIT+CRT (*n* = 55), and CRT + IT (*n* = 132). The median follow‐up time was 31.5 months. Before and after PSM, PFS, and OS were longer in NCIT+Surgery than in CRT + IT, whereas no significant difference was observed between NCIT+Surgery and NCIT+CRT. Subgroup analysis showed that survival did not differ significantly between NCIT+Surgery and CRT + IT in patients with multistation or bulky N2 metastasis, whereas CRT + IT was associated with inferior PFS and OS in patients without these features.

**Conclusions:**

Survival did not differ significantly between CRT and surgery after NCIT, nor between CRT + IT and NCIT + Surgery among patients with multistation or bulky N2 disease. Whether CRT can serve as an alternative to surgery in this population requires validation in large‐scale prospective studies.

**Abbreviations**
ALK: anaplastic lymphoma kinase
CRT: concurrent chemoradiotherapy
CT: computed tomography
CTCAE: Common Terminology Criteria for Adverse Events
ECOG: Eastern Cooperative Oncology Group
EGFR: epidermal growth factor receptor
ICIs: immune checkpoint inhibitors
IT: immunotherapy
MRI: magnetic resonance imaging
NCCN: National Comprehensive Cancer Network
NCIT: neoadjuvant chemoimmunotherapy
NSCLC: non–small cell lung cancer
OS: overall survival
PD‐1: programmed death 1
PD‐L1: programmed death ligand 1
PET‐CT: Positron Emission Computed Tomography
PFS: progression‐free survival
PSM: propensity score matching
ROS1: c‐ros oncogene 1
TRAEs: treatment‐related adverse events

## 1 Introduction

Lung cancer is the most prevalent and fatal malignancy in the world, with non–small cell lung cancer (NSCLC) accounting for approximately 85% [1] of all lung cancer histological subtypes. Approximately 30%–40% of NSCLC cases are resectable or potentially resectable at initial diagnosis [2]. Among them, Stage III–N2 disease exhibits substantial prognostic variability owing to its high heterogeneity in anatomical extent, biological behavior, and treatment response, making the development of optimal treatment strategies particularly challenging and highly individualized. Recent clinical trials, such as CheckMate‐816 [3], AEGEAN [4], KEYNOTE‐671 [5], and CheckMate‐77 T [6], have demonstrated that the addition of programmed death 1 (PD‐1) or programmed death ligand 1 (PD‐L1) inhibitors to neoadjuvant chemotherapy significantly improves the pathological complete response rates and event‐free survival (EFS) in potentially resectable NSCLC. This has prompted the modification of the treatment strategies for Stage III–N2 NSCLC. For potentially resectable T1–3N2 NSCLC, the National Comprehensive Cancer Network (NCCN) guidelines [7] recommend either definitive concurrent chemoradiotherapy (CRT, Category 1 recommendation) or neoadjuvant chemoimmunotherapy (NCIT) with or without radiation, followed by surgery (Category 2A evidence); however, the guidelines do not specify the selection criteria for these approaches. Additionally, in real‐world clinical practice, some patients with potentially resectable Stage III–N2 NSCLC who initially received NCIT were subsequently assessed as inoperable or declined surgery; hence, they underwent definitive CRT instead.

Previous studies have been designed to compare the outcomes of consolidation immunotherapy after definitive CRT vs. surgery following NCIT [8], as well as surgery vs. definitive CRT administered after NCIT [9] in patients with potentially resectable Stage III–N2 NSCLC. These studies demonstrated that the overall survival (OS) was significantly better in patients who underwent surgery after NCIT than in those who received definitive concurrent CRT followed by immunotherapy, and no significant differences in progression‐free survival (PFS) or OS were observed between patients who underwent surgery and definitive CRT following NCIT. However, the comprehensive study that systematically compares these three treatment regimens—NCIT followed by surgery (NCIT+Surgery), NCIT followed by chemoradiotherapy (NCIT+CRT), and definitive concurrent CRT followed by immunotherapy (CRT + IT)—for potentially resectable Stage III–N2 NSCLC is lacking. Moreover, optimal treatment decision‐making remains unclear for specific subgroups of patients with III–N2 NSCLC that exhibit marked differences in prognosis.

Therefore, in this study, we retrospectively included patients with Stage III–N2 NSCLC who received one of three treatment strategies: NCIT+Surgery, NCIT+CRT, or CRT + IT. We aimed to compare these three distinct strategies and to explore whether CRT could represent a feasible option to surgery, thereby providing insights into individualized treatment decision‐making for potentially resectable Stage III–N2 NSCLC in the era of immunotherapy.

## 2 Methods

### 2.1 Patient Selection

This study included 363 patients with potentially resectable Stage III–N2 NSCLC who were treated at Shandong Cancer Hospital and Qilu Hospital of Shandong University between 2019 and 2024. The inclusion criteria were as follows (1): aged between 18 and 75 years (2); histologically confirmed NSCLC according to the World Health Organization (WHO) criteria [10] (3); Stage cT1–3N2M0 (assessed according to the 9th edition of the International Association for the Study of Lung Cancer Thoracic Oncology Staging Manual [11]) (4); the multidisciplinary team (MDT) assessed the disease as resectable or potentially resectable (5); received NCIT followed by either surgery or CRT, or received definitive concurrent CRT followed by consolidation immunotherapy (6); Eastern Cooperative Oncology Group (ECOG) performance status of 0 or 1, with predicted pulmonary function sufficient to tolerate the planned surgical procedure and no unacceptable anesthetic or perioperative risk. The exclusion criteria included (1): mutations in the epidermal growth factor receptor (EGFR), anaplastic lymphoma kinase (ALK), or c‐ros oncogene 1 (ROS1) driver genes (2); histologically confirmed small cell or neuroendocrine lung cancer (3); the presence of other primary malignancies (4); disease progression after NCIT or definitive CRT. Demographic characteristics and therapeutic data were extracted from the electronic medical records. This study was approved by the Institutional Review Board of Shandong Cancer Hospital and the Institute and Qilu Hospital of Shandong University, and was conducted per the Declaration of Helsinki. The requirement for written informed consent for the use of the electronic medical records was waived.

### 2.2 Criteria for Resectability

For Stage III–N2 disease, there is indeed no universally accepted and strictly quantified definition of “resectability” at present. Existing expert consensus statements [12] indicate that, in Stage III–N2 driver gene–negative NSCLC, resectability is generally limited to patients with an anatomically resectable, non‐invasive T1–3 primary tumor and non‐bulky, single‐station N2 involvement, as determined by MDT evaluation. In contrast, the criteria for resectability in patients with invasive T3 disease, bulky N2, or multistation N2 involvement remain highly controversial. All patients included in this study from the two centers underwent MDT discussion prior to treatment. For patients with an anatomically resectable, non‐invasive T1–3 primary tumor and non‐bulky, single‐station N2 involvement, NCIT was recommended, followed by re‐evaluation of surgical indications by thoracic surgeons after completion of treatment. In this study, bulky N2 was defined as a single mediastinal lymph node with a short‐axis diameter ≥ 2 cm, or multiple mediastinal lymph nodes fused into a mass. For patients with T1–3 disease and bulky N2 disease without radiologic evidence of fixation or extranodal extension and with a performance status sufficient to tolerate surgery, the MDT assessed the disease as potentially resectable and recommended neoadjuvant therapy as the initial approach; otherwise, the disease was directly assessed as unresectable. Patients with T1–3 disease and multistation N2 involvement were likewise considered potentially resectable by the MDT and were recommended to receive neoadjuvant therapy first. After completion of neoadjuvant therapy, patients with bulky or multistation N2 disease were required to have true mediastinal downstaging pathologically confirmed by bronchoscopy/endobronchial ultrasonography (EBUS), defined as downstaging from bulky N2 to N0 or N1, or to single‐station, non‐bulky N2. Thoracic surgeons then re‐evaluated resectability; patients who met the operative criteria proceeded to surgical resection. For invasive T3 lesions, if the MDT determined that the disease was localized, could be completely resected, and would not result in an unacceptable increase in surgical risk, neoadjuvant therapy was recommended, followed by reassessment of surgical indications after treatment.

### 2.3 Treatment

Patients were categorized into three groups based on the treatment modality: [1] NCIT+Surgery; [2] NCIT+CRT; and [3] CRT + IT. The NCIT regimen comprised two to four cycles of platinum‐based doublet chemotherapy combined with PD‐1 or PD‐L1 inhibitors [13, 14, 15, 16], administered every 3 weeks. For non‐squamous NSCLC, chemotherapy consisted of cisplatin or carboplatin plus pemetrexed or a taxane; for squamous NSCLC, cisplatin or carboplatin was administered in combination with a taxane. PD‐1/PD‐L1 inhibitors (such as pembrolizumab, nivolumab, durvalumab, camrelizumab, tislelizumab, toripalimab, or sintilimab) were given intravenously on day 1 of each cycle according to the approved dosing schedules. Patients deemed operable underwent radical resection within 4–8 weeks after completing NCIT, whereas those who were ineligible for operation or declined surgery received definitive CRT. Postoperative and consolidation immunotherapies after CRT were also viable options. Additionally, some patients received definitive CRT, followed by consolidation with PD‐1/PD‐L1 inhibitors. Intensity‐modulated radiotherapy (IMRT) was selected as the radiotherapy technique. The gross tumor volume (GTV [17]) encompassed the primary lung tumor and the mediastinal or hilar lymph nodes confirmed to be metastatic via contrast‐enhanced computed tomography (CT) or Positron Emission Computed Tomography (PET‐CT). The clinical target volume (CTV) was defined as the expansion of the GTV by 6 mm, including the hilar and mediastinal lymphatic drainage regions. The planning target volume (PTV) was generated by adding a 5‐mm margin to the CTV. The prescribed radiation dose ranged from 54 to 66 Gy and was delivered once daily, 5 days per week.

### 2.4 Follow‐Up and Study Endpoints

The follow‐up period extended from treatment initiation to the final follow‐up cut‐off date (May 31, 2025) or the patient's death. Follow‐up evaluations were performed at 3‐month intervals during the first 2 years after surgery or CRT, using brain magnetic resonance imaging (MRI) or CT, and contrast‐enhanced chest CT. After the initial 2 years, the follow‐up intervals were extended to 6 months. The treatment response was evaluated according to the Response Evaluation Criteria in Solid Tumors version 1.1 (RECIST v1.1 [18]). The primary endpoints were PFS and OS. PFS was defined as the interval from the initial treatment to the first occurrence of disease progression, and OS was defined as the interval from the initial treatment to death from any cause. Treatment‐related adverse events (TRAEs) in individual patients were assessed using the Common Terminology Criteria for Adverse Events (CTCAE), version 5.0 [19].

### 2.5 Statistical Analysis

Fisher's exact test or the chi‐square test was performed to determine the differences in nominal categorical variables. The Kaplan–Meier method was used to estimate follow‐up time and survival curves, and univariate analysis was conducted using the log‐rank test. Propensity score matching (PSM) was performed to control for confounding factors between groups. Propensity scores for the NCIT+Surgery and NCIT+CRT groups were calculated using a logistic regression model that included covariates such as sex, age, pathology, stage, PD‐L1 expression levels, radiological response, and ECOG performance status. Propensity scores for NCIT+Surgery and CRT + IT groups were calculated using a logistic regression model that included covariates such as sex, age, pathology, ECOG performance status, stage, and PD‐L1 expression levels. Based on the estimated propensity scores, a caliper width of 0.02 was used to apply caliper matching and achieve a 1:1 match between the two groups. Statistical significance was set at *p* < 0.05. All statistical analyses were performed using the SPSS software (version 27.0, IBM Corporation), and survival curves were created using Prism software (version 10.1.2, GraphPad).

## 3 Results

### 3.1 Baseline Characteristics

Among the 363 patients diagnosed with cT1–3N2M0 NSCLC who met the eligibility criteria at the two institutions, 176 patients received NCIT+Surgery, 55 underwent NCIT+CRT, and 132 patients received CRT + IT. The median age of patients in the NCIT+Surgery, NCIT+CRT, and CRT + IT groups was 65 years. In the NCIT+Surgery group, 88.6% of the patients were male, compared with 87.3% and 90.9% in the NCIT+CRT and CRT + IT groups, respectively. Squamous cell carcinoma was diagnosed in 71.6%, 70.9%, and 76.5% of patients in the NCIT+Surgery, NCIT+CRT, and CRT + IT groups, respectively. The detailed baseline characteristics of the three groups and those of the two centers are presented in Table 1 and Table S1, respectively, showing no significant differences observed among them. After a 1:1 PSM, the NCIT+Surgery and the NCIT+CRT groups each included 54 patients, whereas the NCIT+Surgery and the CRT + IT groups each included 105 patients. The baseline characteristics of the groups after PSM are shown in Tables S2 and S3.

**TABLE 1:** Baseline characteristics of the entire patient cohort.

	NCIT + Surgery (*n* = 176)	NCIT + CRT (*n* = 55)	CRT + IT (*n* = 132)	*P* Value
Age (years)	65 (58–68)	65 (60–70)	65 (60–70)	0.815
Gender				0.715
Male	156 (88.6%)	48 (87.3%)	120 (90.9%)	
Female	20 (11.4%)	7 (12.7%)	12 (9.1%)	
Stage				0.595
IIIA	69 (39.2%)	19 (34.5%)	56 (42.4%)	
IIIB	107 (60.8%)	36 (65.5%)	76 (57.6%)	
Smoking history				0.576
No	45 (25.6%)	13 (23.6%)	27 (20.5%)	
Yes	131 (74.4%)	42 (76.4%)	105 (79.5%)	
Histology				0.571
Squamous	126 (71.6%)	39 (70.9%)	101 (76.5%)	
Non‐squamous	50 (28.4%)	16 (29.1%)	31 (23.5%)	
Metastatic N2 lymph node status				0.446
N2a	69 (39.2%)	22 (40.0%)	61 (46.2%)	
N2b	107 (60.8%)	33 (60.0%)	71 (53.8%)	
Bulky N2 disease				0.650
No	123 (69.9%)	38 (69.1%)	98 (74.2%)	
Yes	53 (30.1%)	17 (30.9%)	34 (25.8%)	
PD‐L1 expression level				0.808
1%	68 (38.7%)	21 (38.2%)	51 (38.6%)	
≥ 1%	84 (47.7%)	29 (52.7%)	68 (51.5%)	
Unknown	24 (13.6%)	5 (9.1%)	13 (9.9%)	
ECOG performance status				0.854
0	57 (32.4%)	18 (32.7%)	43 (32.6%)	
1	116 (65.9%)	37 (67.3%)	88 (66.6%)	
2	3 (1.7%)	0 (0.0%)	1 (0.8%)	

Abbreviations: CRT, chemoradiotherapy; ECOG, Eastern Cooperative Oncology Group; IT, immunotherapy; NCIT, neoadjuvant chemoimmunotherapy; PD‐L1, programmed death ligand 1.

### 3.2 Survival Analysis in the Whole Population

**FIGURE 1:**
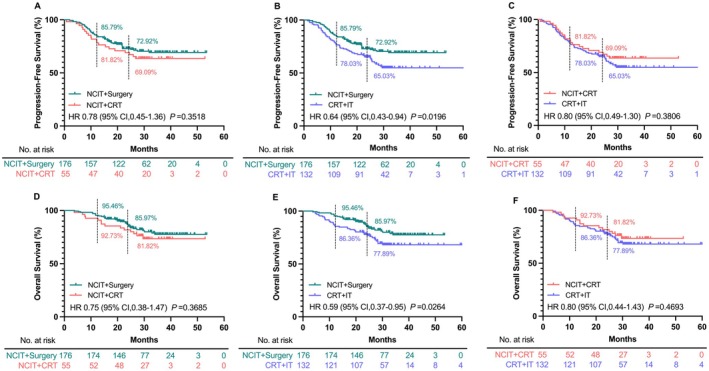
PFS (A‐C) and OS (D‐F) in the NCIT+Surgery, NCIT+CRT, and CRT + IT groups in the entire patient cohort. (A) NCIT+Surgery and NCIT+CRT groups; (B) NCIT+Surgery and CRT + IT groups; (C) NCIT+CRT and CRT + IT groups; (D) NCIT+Surgery and NCIT+CRT groups; (E) NCIT+Surgery and CRT + IT groups; (F) NCIT+CRT and CRT + IT groups. Abbreviation: PFS, progression‐free survival; OS, overall survival; NCIT, neoadjuvant chemoimmunotherapy; CRT, chemoradiotherapy; IT, immunotherapy.

The median follow‐up period for the entire cohort was 31.5 months. There were no significant differences in PFS between the NCIT+Surgery and NCIT+CRT groups, or between the NCIT+CRT and CRT + IT groups (Figure 1A,C). However, compared with that in the CRT + IT group, the NCIT+Surgery group showed a significantly improved PFS (HR: 0.64, 95% CI: 0.43–0.94, *p* = 0.0196; Figure 1B). The OS results were consistent with those of PFS, with no significant differences between the NCIT+Surgery and NCIT+CRT groups (Figure 1D) or between NCIT+CRT and CRT + IT groups (Figure 1F). Conversely, a significant OS benefit was observed in the NCIT+Surgery group compared with that in the CRT + IT group (HR: 0.59, 95% CI: 0.37–0.95, *p* = 0.0264; Figure 1E).

### 3.3 Survival Analysis After PSM Between the NCIT+Surgery and NCIT+CRT Groups

**FIGURE 2:**
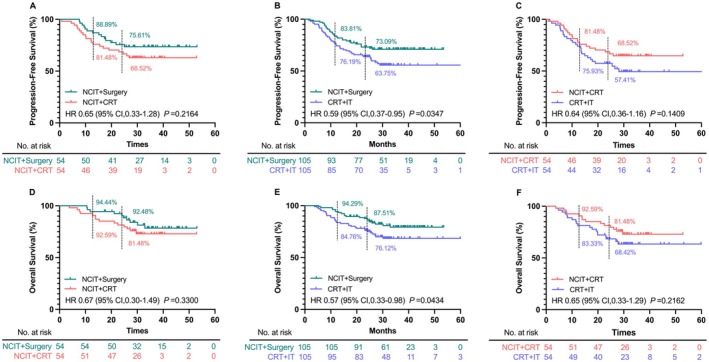
PFS (A‐C) and OS (D‐F) in the NCIT+Surgery, NCIT+CRT, and CRT + IT groups after PSM. (A) NCIT+Surgery and NCIT+CRT groups; (B) NCIT+Surgery and CRT + IT groups; (C) NCIT+CRT and CRT + IT groups; (D) NCIT+Surgery and NCIT+CRT groups; (E) NCIT+Surgery and CRT + IT groups; (F) NCIT+CRT and CRT + IT groups. Abbreviation: PFS, progression‐free survival; OS, overall survival; NCIT, neoadjuvant chemoimmunotherapy; CRT, chemoradiotherapy; IT, immunotherapy; PSM, propensity score matching.

**FIGURE 3:**
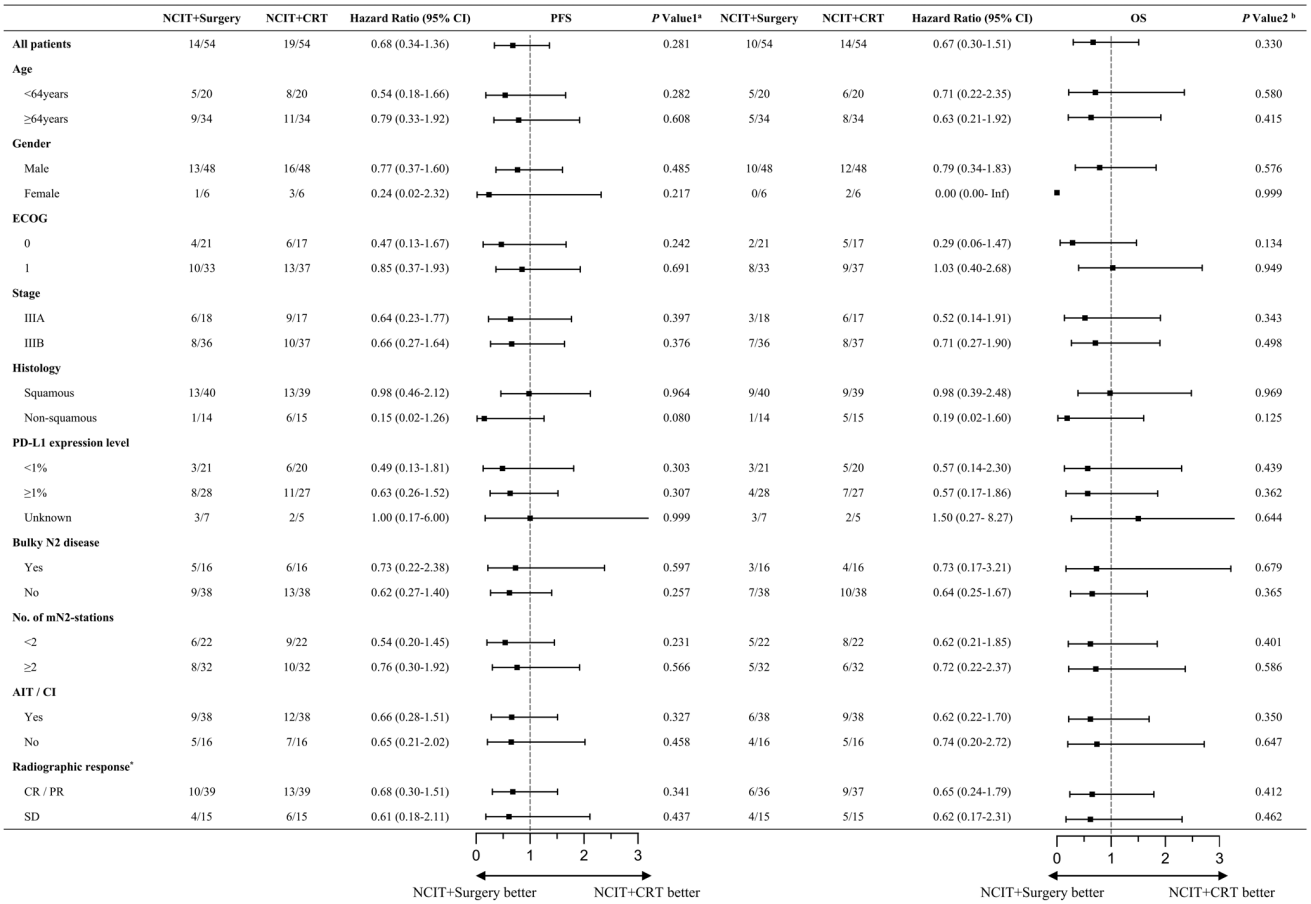
Forest plot of PFS and OS comparing NCIT+Surgery versus NCIT+CRT groups across subgroups after 1:1 PSM. Abbreviations: PFS, progression‐free survival; OS, overall survival; NCIT, neoadjuvant chemoimmunotherapy; CRT, chemoradiotherapy; IT, immunotherapy; PSM, propensity score matching; CI, confidence interval; PD‐L1, programmed death ligand 1; CR, complete response; PR, partial response; SD, stable disease. * Radiographic response to NCIT according to RECIST criteria. ^a^ *P* value 1 represents the *P* value for PFS between the NCIT+Surgery and NCIT+CRT groups after PSM. ^b^ *P* value 2 represents the *P* value for OS between the NCIT+Surgery and NCIT+CRT groups after PSM.

After a 1:1 PSM between the NCIT+Surgery and the NCIT+CRT groups, the PFS and OS results remained consistent with those observed before matching. The PFS did not differ significantly between the NCIT+CRT and NCIT+Surgery groups (Figure 2A). The 1‐ and 2‐year PFS rates were 88.89% and 75.61% in the NCIT+Surgery group, and 81.48% and 68.52% in the NCIT+CRT group, respectively. Similarly, the OS exhibited no significant improvement in the NCIT+Surgery group compared with that in the NCIT+CRT group (Figure 2D). The 1‐ and 2‐year OS rates were 94.44% and 92.48% in the NCIT+Surgery group, and 92.59% and 81.48% in the NCIT+CRT group, respectively. Further subgroup analyses similarly demonstrated no significant PFS or OS benefit across patient baseline characteristics (Figure 3).

### 3.4 Survival Analysis After PSM Between the NCIT+Surgery and CRT + IT Groups

**FIGURE 4:**
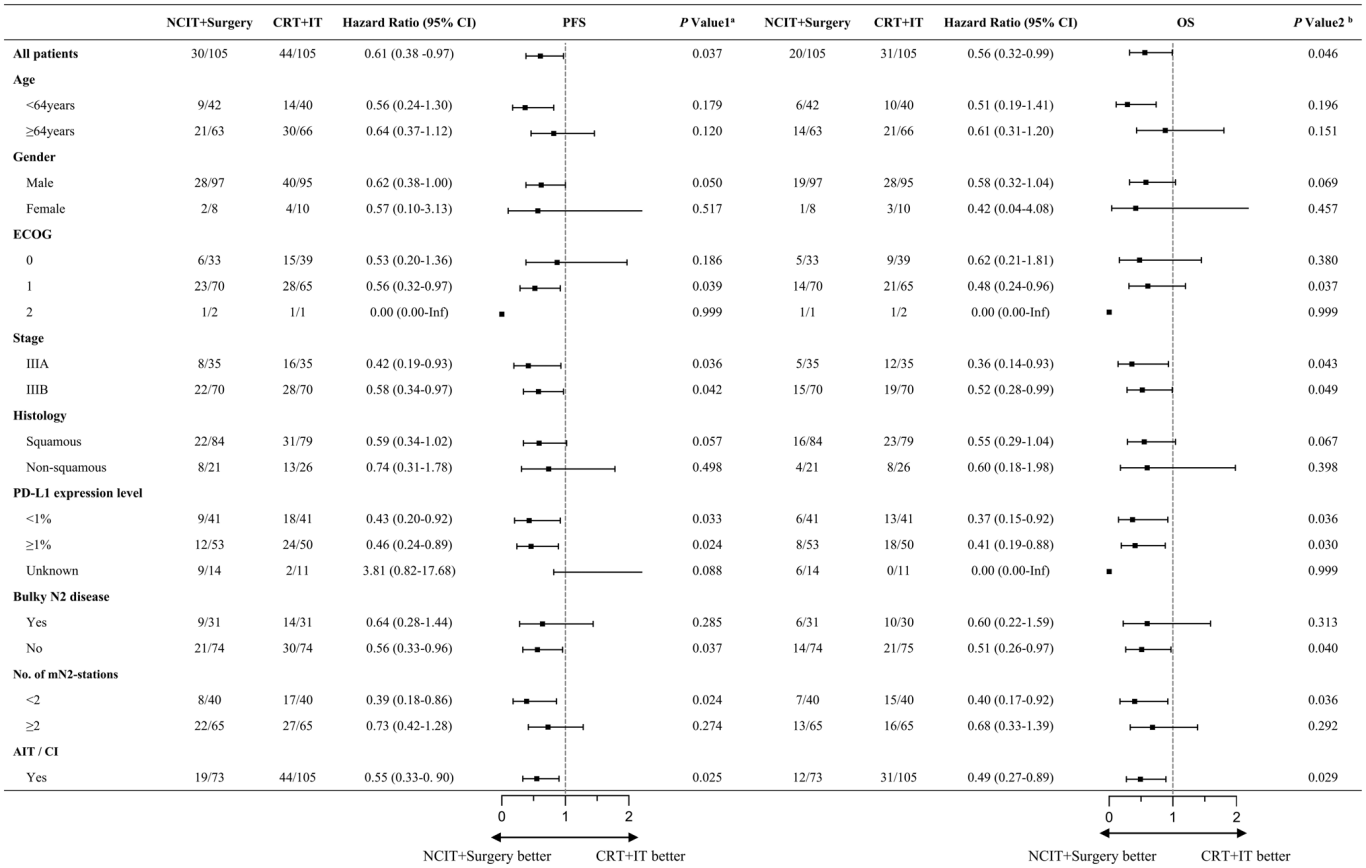
Forest plot of PFS and OS comparing NCIT+Surgery versus CRT + IT groups across subgroups after 1:1 PSM. Abbreviations: PFS, progression‐free survival; OS, overall survival; NCIT, neoadjuvant chemoimmunotherapy; CRT, chemoradiotherapy; IT, immunotherapy; PSM, propensity score matching; CI, confidence interval; PD‐L1, programmed death ligand 1. ^a^ *P* value 1 represents the *P* value for PFS between the NCIT+Surgery and CRT + IT groups after PSM. ^b^ *P* value 2 represents the *P* value for OS between the NCIT+Surgery and CRT + IT groups after PSM.

Following a 1:1 PSM between the NCIT+Surgery and CRT + IT groups, the PFS and OS outcomes remained consistent with the pre‐matching results. The NCIT+Surgery group showed a significant improvement in PFS compared with that in the CRT + IT group (HR: 0.59, 95% CI: 0.37–0.95, *p* = 0.0347; Figure 2B). The 1‐ and 2‐year PFS rates in the NCIT+Surgery group were 83.81% and 73.09%, respectively, while those in the CRT + IT group were 76.19% and 63.75%, respectively. Similarly, regarding OS, the NCIT+Surgery group demonstrated a significant improvement compared to the NCIT+CRT group (HR: 0.57, 95% CI: 0.33–0.98, *p* = 0.0434; Figure 2E), with 1‐ and 2‐year OS rates of 94.29% and 87.51% in the NCIT+Surgery group, and 84.76% and 76.12% in the CRT + IT group, respectively. Subgroup analyses showed that, across most baseline characteristics, NCIT+Surgery tended to confer a survival benefit over CRT + IT (Figure 4).

### 3.5 Safety

We further compared the incidence of common TRAEs between each matched pair of groups after PSM. TRAEs occurred in 50 (92.6%) vs. 48 (88.9%) patients in the NCIT+Surgery and NCIT+CRT groups, in 94 (89.5%) vs. 97 (92.4%) patients in the NCIT+Surgery and CRT + IT groups, and in 47 (87.0%) vs. 48 (88.9%) patients in the NCIT+CRT and CRT + IT groups. No significant differences in either all TRAEs or Grade ≥ 3 TRAEs were observed among the three groups. Hematological toxicity was the most common TRAE in the three groups. The NCIT+Surgery group had a significantly lower incidence of Grade ≥ 3 hematologic toxicity than did the NCIT+CRT and CRT + IT groups (NCIT+Surgery vs. NCIT+CRT: 11.1% vs. 27.8%, *p* = 0.029; NCIT+Surgery vs. CRT + IT: 21.8% vs. 31.4%, *p* < 0.001), whereas no significant difference was observed between the NCIT+CRT and CRT + IT groups. The NCIT+Surgery group experienced significantly fewer all‐grade pneumonitis events than did the NCIT+CRT or CRT + IT groups, with incidences of 13.0% vs. 31.5% (*p* = 0.021) and 19.0% vs. 34.3% (*p* = 0.013), respectively; however, the incidence of Grade ≥ 3 pneumonitis did not differ significantly among the three groups. Moreover, compared with those in the other two groups, the NCIT+Surgery group also showed a statistically significant reduction in the incidence of all‐grade esophagitis and gastrointestinal disorder adverse events; however, no Grade ≥ 3 esophagitis or gastrointestinal disorders were observed in the three groups. Details of the safety profiles are presented in Table 2.

**TABLE 2:** TRAEs across the NCIT+Surgery, NCIT+CRT and CRT + IT groups of the entire patient cohort.

Adverse events	After PSM		*P* Value1^a^	After PSM		*P* Value2^b^	After PSM		*P* Value3^c^
	NCIT + Surgery (*n* = 54)	NCIT + CRT (*n* = 54)		NCIT + Surgery (*n* = 105)	CRT + IT (*n* = 105)		NCIT + CRT (*n* = 54)	CRT + IT (*n* = 54)	
Treatment‐related AEs	50 (92.6%)	48 (88.9%)	0.507	94 (89.5%)	97 (92.4%)	0.470	47 (87.0%)	48 (88.9%)	0.767
G ≥ 3 treatment‐related AEs	23 (42.6%)	26 (48.1%)	0.562	53 (50.5%)	59 (56.2%)	0.407	28 (51.9%)	26 (48.1%)	0.700
All grade hematologic toxicity	35 (64.8%)	38 (70.4%)	0.537	76 (72.4%)	82 (78.1%)	0.337	40 (74.1%)	38 (70.4%)	0.667
G ≥ 3 hematologic toxicity	6 (11.1%)	15 (27.8%)	0.029	12 (21.8%)	33 (31.4%)	< 0.001	12 (22.2%)	10 (18.5%)	0.633
All grade pneumonitis	7 (13.0%)	17 (31.5%)	0.021	20 (19.0%)	36 (34.3%)	0.013	14 (25.9%)	13 (24.1%)	0.824
G ≥ 3 pneumonitis	1 (1.9%)	3 (5.6%)	0.610	3 (2.9%)	5 (4.8%)	0.718	3 (5.6%)	2 (3.7%)	1.000
All grade esophagitis	0 (0.0%)	14 (25.9%)	< 0.001	0 (0.0%)	22 (21.0%)	< 0.001	13 (24.1%)	12 (22.2%)	0.820
G ≥ 3 esophagitis	0 (0.0%)	0 (0.0%)	—	0 (0.0%)	0 (0.0%)	—	0 (0.0%)	0 (0.0%)	—
Gastrointestinal disorder	0 (0.0%)	6 (11.1%)	0.036	0 (0.0%)	10 (9.5%)	0.001	7 (13.0%)	6 (11.1%)	0.767
G ≥ 3 gastrointestinal disorder	0 (0.0%)	0 (0.0%)	—	0 (0.0%)	0 (0.0%)	—	0 (0.0%)	0 (0.0%)	—
Hepatic function damage	3 (5.6%)	4 (7.4%)	1.000	8 (7.6%)	7 (6.7%)	0.789	4 (7.4%)	2 (3.7%)	0.674
G ≥ 3 hepatic function damage	0 (0.0%)	1 (1.9%)	1.000	2 (1.9%)	1 (1.0%)	1.000	1 (1.9%)	1 (1.9%)	1.000
Surgery‐related AEs	23 (42.6%)	0 (0.0%)	< 0.001	43 (41.0%)	0 (0.0%)	< 0.001	0 (0.0%)	0 (0.0%)	—
G ≥ 3 Surgery‐related AEs	5 (9.3%)	0 (0.0%)	0.067	10 (9.5%)	0 (0.0%)	0.004	0 (0.0%)	0 (0.0%)	—

Abbreviations: CRT, chemoradiotherapy; IT, immunotherapy; NCIT, neoadjuvant chemoimmunotherapy.

^a^ *P* value 1 represents the *P* value between the NCIT+Surgery and NCIT+CRT groups after PSM.

^b^ *P* value 2 represents the *P* value between the NCIT+Surgery and CRT + IT groups after PSM.

^c^ *P* value 3 represents the *P* value between the NCIT+CRT and CRT + IT groups after PSM.

### 3.6 Subgroup Analysis of Metastatic N2 Lymph Node Status

Given the considerable heterogeneity of Stage III–N2 NSCLC and the varying prognoses associated with different forms of N2 lymph node involvement, a subgroup analysis was conducted according to the N2 status. After PSM, among patients with multistation (≥ 2) or bulky N2 metastases, there were no significant differences in the PFS between the NCIT+Surgery and CRT + IT groups (HR: 0.73, 95% CI: 0.42–1.28, *p* = 0.2740; Figure S1F; HR: 0.64, 95% CI: 0.28–1.44, *p* = 0.2849; Figure S2F). Conversely, in patients without multistation or bulky N2 metastases, the CRT + IT group exhibited significantly inferior PFS compared with that of the NCIT+Surgery group (HR: 0.39, 95% CI: 0.18–0.88, *p* = 0.0237; Figure S1B; HR: 0.56, 95% CI: 0.33–0.96, *p* = 0.0366; Figure S2B). A similar trend was observed for OS. In patients with multistation or bulky N2 metastases, OS showed no differences between the two groups (HR: 0.68, 95% CI: 0.33–1.39, *p* = 0.2915; Figure S1H; HR: 0.60, 95% CI: 0.22–1.59, *p* = 0.3128; Figure S2H). However, in patients without multistation or bulky N2 involvement, the NCIT+Surgery group showed a significant OS benefit (HR: 0.40, 95% CI: 0.17–0.92, *p* = 0.0364; Figure S1D; HR: 0.51, 95% CI: 0.26–0.97, *p* = 0.0396; Figure S2D).

### 3.7 Subgroup Analysis of PD‐L1 Expression Levels

In the subgroup analysis based on PD‐L1 expression levels, the NCIT+Surgery group showed no significant difference in either PFS or OS compared with those in the NCIT+CRT group, regardless of whether PD‐L1 expression was < 1% or ≥ 1%. However, compared with those in the CRT + IT group, the NCIT+Surgery group demonstrated significant benefits in both PFS (HR: 0.43, 95% CI: 0.20–0.92, *p* = 0.0328; Figure S3E) and OS (HR: 0.37, 95% CI: 0.15–0.92, *p* = 0.0361; Figure S3G) among patients with PD‐L1 < 1%. In addition, in those with PD‐L1 ≥ 1%, the NCIT+Surgery group also showed significant benefits in PFS (HR: 0.46, 95% CI: 0.24–0.89, *p* = 0.0244; Figure S3F) and OS (HR: 0.41, 95% CI: 0.19–0.88, *p* = 0.0297; Figure S3H).

### 3.8 Subgroups Analysis of Stage, Radiographic Response, and Adjuvant Immunotherapy

In the stage‐based subgroup analysis, the results were consistent with those of the overall population (Figure S4). Treatment response to NCIT was also evaluated. Regardless of whether patients showed a radiological response to NCIT, the NCIT+Surgery group did not demonstrate significant benefits in either PFS or OS compared with those in the NCIT+CRT group (Figure S5). Similarly, in the adjuvant immunotherapy subgroup analysis, the NCIT+Surgery group showed no apparent trend toward improved PFS or OS compared with those in the NCIT+CRT group, with or without the administration of adjuvant immunotherapy (Figure S6A–D). Among patients receiving adjuvant immunotherapy, the NCIT+CRT group showed no significant benefits in either PFS or OS compared with those in the CRT + IT group (Figure S6G, H). In contrast, among patients who received adjuvant immunotherapy, the NCIT+Surgery group demonstrated significant benefits in both PFS (HR: 0.55, 95% CI: 0.33–0.90, *p* = 0.0253; Figure S6E) and OS (HR: 0.49, 95% CI: 0.27–0.89, *p* = 0.0292; Figure S6F) compared with those in the CRT + IT group.

## 4 Discussion

In this multi‐institutional retrospective study, we evaluated different strategies for potentially resectable Stage III–N2 NSCLC, and found that the NCIT+Surgery group achieved significantly improved PFS and OS compared with those in the CRT + IT group. However, among patients with bulky or multistation N2 metastases, no significant differences in PFS or OS were observed between the NCIT+Surgery and CRT + IT groups. Moreover, the PFS and OS benefits showed no significant differences between the NCIT+Surgery and NCIT+CRT groups in the overall cohort or in any subgroup. Overall, our real‐world data indicate that survival did not differ significantly between CRT and surgery following NCIT, nor between CRT + IT and NCIT+Surgery in patients with bulky or multistation N2 disease. Given the limited sample size, these findings should be interpreted with caution and considered exploratory and hypothesis‐generating rather than confirmatory. Nevertheless, they may offer preliminary insights and help inform the design of future adequately powered prospective trials.

Treatment for potentially resectable, driver gene–negative Stage III–N2 NSCLC is shifting from traditional neoadjuvant chemotherapy with or without radiotherapy to NCIT. Current guidelines [7] recommend two primary strategies: Surgery following NCIT or definitive CRT, with the choice determined based on multidisciplinary evaluation and operability. However, the optimal indications for surgery vs. CRT remain unclear in the context of a highly heterogeneous N2 disease burden. Previous retrospective studies [8, 20] have demonstrated that NCIT, followed by surgery, provides significantly superior PFS and OS compared with those in CRT + IT, consistent with our findings.

We conducted the first subgroup analysis stratified by N2 lymph node status and found consistent results with those of the overall analysis in patients without bulky or multistation N2 metastases. In contrast, in patients with bulky or multistation N2 disease, no significant differences in PFS or OS were observed between the two groups (NCIT + Surgery and CRT + IT). Given the distinct biological and clinical characteristics of these two patterns, we further discussed them separately. On the one hand, multistation N2 disease reflects extensive nodal involvement and a high risk of occult systemic micrometastases, indicating more aggressive tumor biology. In this setting, long‐term outcomes are less dependent on intensified local control and more on sustained systemic disease suppression. Accordingly, the incremental local tumor reduction achieved through surgery may have a limited impact on overall survival. By contrast, CRT + IT offers established locoregional control while allowing for continued immune‐mediated suppression of micrometastatic disease, which may, at least in part, account for the comparable survival outcomes observed between the two treatment approaches. On the other hand, bulky N2 disease is characterized by a high mediastinal tumor burden and relative resistance to treatment. Even when R0 resection is technically achievable following NCIT, the complete eradication of microscopic residual mediastinal disease can remain challenging, which could potentially attenuate the long‐term survival benefit of surgery. In comparison, definitive CRT permits more comprehensive coverage of mediastinal disease, and the addition of consolidation immunotherapy may further contribute to improved locoregional control and sustained systemic antitumor immune activity, possibly through mechanisms related to radiotherapy‐induced immunogenic cell death. These factors may partly account for the lack of a clear survival advantage of NCIT + surgery over CRT + IT in patients with bulky N2 disease. However, it should be noted that the sample sizes of the bulky N2 and multistation N2 subgroups in this study were limited, and therefore, the biological and clinical significance of these results should be interpreted with caution.

The prognostic differences between surgery and radiotherapy after NCIT remain controversial. Li and Guan et al. [9, 20] reported no significant differences in PFS or OS between surgery after NCIT and radiotherapy/CRT following NCIT before and after PSM. In contrast, Qi et al. [21] found that survival in the surgery group was significantly superior to that in the radiotherapy group regardless of PSM adjustment. Several factors may influence the discrepancies between the studies. First, differences in patient selection and baseline characteristics, such as tumor burden [22] and extent of nodal involvement, may influence the relative benefits of surgery vs. radiotherapy. Second, variations in treatment implementation, including the extent of lymph node dissection, radiotherapy protocols, and the use of adjuvant or consolidation immunotherapy, may affect the outcomes [23, 24]. Third, the assessment of NCIT response and subsequent multidisciplinary decision‐making are subjective [25], with patients showing marked tumor regression or better overall condition being more likely to undergo surgery, introducing a selection bias. Finally, the relatively short follow‐up periods in some studies may have caused the underestimation of long‐term survival differences. Our results indicated no significant difference in survival between patients who underwent surgery and those who received CRT after NCIT. However, given the relatively small sample size, this finding should be interpreted with caution. To address concerns regarding whether the available sample size was sufficient to detect clinically meaningful differences, we conducted a post hoc event‐driven assessment based on the Schoenfeld/log‐rank framework (two‐sided α = 0.05, 1:1 allocation), which is appropriate for time‐to‐event analyses and is primarily determined by the number of observed events. In the PSM cohort (NCIT+Surgery vs. NCIT+CRT, 54 vs. 54), the median follow‐up was 33.8 months. A total of 34 EFS events were observed (14 vs. 20). The log‐rank test showed no statistically significant difference (*p* = 0.216), with an HR of 0.65 (95% CI, 0.33–1.28). An absolute improvement of 10% in the 2‐year EFS rate was prespecified as a clinically meaningful difference; given an estimated 2‐year EFS of approximately 69% in the NCIT+CRT group, this corresponds to a target HR of approximately 0.64 under a proportional hazards assumption. With 34 observed events, the achieved statistical power to detect such an effect size was approximately 26%. Under the same assumptions, approximately 158 EFS events would be required to achieve 80% power to detect an effect of this magnitude, whereas the current dataset would only have 80% power to detect a substantially larger difference (minimum detectable HR≈0.38). These results indicate that the EFS comparison is limited by the number of observed events, leading to wide confidence intervals and limited precision. Given the above results, we consider that non‐significant *P* values observed in analyses with limited group sizes should not be interpreted as evidence of equivalence or absence of a true difference, and the observed non‐significant differences between the NCIT+Surgery and NCIT+CRT groups should be regarded as inconclusive and hypothesis‐generating rather than as evidence of comparable efficacy. Therefore, prospective randomized controlled trials are warranted to further clarify prognostic differences and to help identify the optimal candidates for surgery vs. definitive CRT after NCIT.

In the NCIT+CRT and CRT + IT groups, no significant differences in PFS or OS were observed. Several factors could explain this finding. First, both groups received platinum‐based chemotherapy combined with immunotherapy (either induction or consolidation), leading to comparable cumulative immune checkpoint inhibitor (ICI) exposure and chemotherapy intensity, which may have minimized differences in outcomes; second, definitive‐dose radiotherapy was delivered to intrathoracic lesions and involved mediastinal nodes in both groups, possibly achieving similar levels of locoregional control [26]; third, the immunogenic cell death induced by NCIT and the immune‐potentiating effects associated with CRT may provide comparable systemic benefits [27, 28], which are achieved at different treatment time points.

In this study, the three treatment regimens showed somewhat different TRAE patterns; however, the most common TRAEs were largely comparable across the groups [24]. Patients receiving NCIT+CRT or CRT + IT experienced more frequent Grade ≥ 3 hematologic toxicities than did those who underwent NCIT+Surgery, possibly owing to the direct myelosuppressive effects of radiotherapy compounded by chemotherapy [29], whereas immunotherapy has a distinct toxicity profile primarily characterized by immune‐related adverse events with relatively mild bone marrow suppression. Conversely, the NCIT+Surgery group exhibited certain unique surgery‐related Grade ≥ 3 TRAEs compared with those in the other two groups [30]. Pneumonitis is a major safety concern worldwide. It is triggered by ICIs or radiotherapy and usually occurs when the two modalities are concurrently administered [31]. In this study, the NCIT+Surgery group experienced significantly fewer all‐grade pneumonitis events than did the NCIT+CRT or CRT + IT groups. However, the incidence of Grade ≥ 3 pneumonitis did not differ significantly among the three groups. Overall, our study demonstrated that the incidences of total and Grade ≥ 3 TRAEs were comparable among the three groups, which is consistent with previous findings [9, 20, 21] and indicates similar, acceptable safety profiles across the three treatment strategies.

Our study has some limitations. Firstly, this was a retrospective study with inherent selection bias. We used PSM to reduce confounding factors. However, residual bias, including unmeasured factors that influence surgical assessment, cannot be ruled out. Secondly, regimen heterogeneity (types and cycles of ICIs/chemotherapy, radiation therapy dose/fractionation, and adjuvant ICIs duration) could influence the outcomes. Thirdly, although 31.5 months of follow‐up is informative, an extended follow‐up is required to define long‐term OS, particularly in cohorts receiving immunotherapy. Then, differences in operability may exist between the two groups, and PSM could not fully address this bias. Patients who underwent surgery generally had fewer comorbidities and better cardiopulmonary function than did those who received CRT after NCIT. Finally, all patients in the CRT + IT group in this study were patients for whom neoadjuvant therapy had also been recommended by the MDT but who declined neoadjuvant treatment and instead chose definitive CRT followed by sequential immunotherapy consolidation. As a result, this group lacked post‐neoadjuvant reassessment of surgical indications, making it impossible to determine whether they might have become candidates for surgical resection, which may have introduced selection bias and limited the comparability between treatment groups.

In conclusion, among patients with potentially resectable Stage III–N2 driver gene–negative NSCLC, our study compared three treatment strategies—NCIT+Surgery, NCIT+CRT, and CRT + IT. The NCIT+Surgery group demonstrated a significant survival advantage over the CRT + IT, but no significant difference compared with the NCIT+CRT group. Among patients with bulky or multistation N2 metastases, no significant survival differences were observed between the NCIT+Surgery and CRT + IT groups. Given the limited subgroup size of the NCIT+CRT group and the bulky or multistation N2 subgroups, these non‐significant findings should be interpreted as inconclusive and hypothesis‐generating rather than as evidence of comparable efficacy between treatment strategies. Nevertheless, larger prospective studies are warranted to validate these findings and to further define the optimal management strategies for this patient population.

## Author Contributions

Jiarui Zhao: writing – original draft, methodology, formal analysis, data curation. Baiyang Huang: methodology, data curation, conceptualization. Min Li: methodology, data curation. Xingpeng Wang: methodology, data curation. Jingyu Zhu: data curation. Kaiyue Wang: data curation. Jing Xu: data curation. Xiaohan Wang: project administration, methodology. Menglin Bai: data curation. Guoxin Cai: writing – review and editing, visualization, validation, supervision, investigation.

## Funding

This work was supported by the National Natural Science Foundation of China, 82172720, 82403791, 82573453; the National Science and Technology Major Project, 2024ZD0525902; the Natural Science Foundation of Shandong Province, ZR2024QH459; the Shandong Province University “Youth” Innovation Team Program, 2024KJJ027”; the Chinese Society of Clinical Oncology, Y‐2022HER2AZMS‐0291; and the Collaborative Academic Innovation Project of Shandong Cancer Hospital, ZF001.

## Ethics Statement

This study was approved by the Institutional Review Board of Shandong Cancer Hospital and the Institute and Qilu Hospital of Shandong University, and was conducted per the Declaration of Helsinki. The requirement for written informed consent for the use of the electronic medical records was waived.

## Conflicts of Interest

The authors declare no conflicts of interest.

## Supporting information


**FIGURE S1:** PFS and OS across the NCIT+Surgery, NCIT+CRT, and CRT + IT groups, stratified by the number of metastatic mediastinal lymph node stations (< 2 vs. ≥ 2) after PSM. (A) PFS between the NCIT+Surgery and NCIT+CRT groups with < 2 metastatic mediastinal lymph node stations; (B) PFS between the NCIT+Surgery and CRT + IT groups with < 2 metastatic mediastinal lymph node stations; (C) OS between the NCIT+Surgery and NCIT+CRT groups with < 2 metastatic mediastinal lymph node stations; (D) OS between the NCIT+Surgery and CRT + IT groups with < 2 metastatic mediastinal lymph node stations; (E) PFS between the NCIT+Surgery and NCIT+CRT groups with ≥ 2 metastatic mediastinal lymph node stations; (F) PFS between the NCIT+Surgery and CRT + IT groups with ≥ 2 metastatic mediastinal lymph node stations; (G) OS between the NCIT+Surgery and NCIT+CRT groups with ≥ 2 metastatic mediastinal lymph node stations; (H) OS between the NCIT+Surgery and CRT + IT groups with ≥ 2 metastatic mediastinal lymph node stations. Abbreviation: PFS, progression‐free survival; OS, overall survival; NCIT, neoadjuvant chemoimmunotherapy; CRT, concurrent chemoradiotherapy; IT, immunotherapy; PSM, propensity score matching.
**FIGURE S2:** PFS and OS across the NCIT+Surgery, NCIT+CRT, and CRT + IT groups, stratified by bulky N2 status (present or absent) after PSM. (A) PFS between the NCIT+Surgery and NCIT+CRT groups without bulky N2; (B) PFS between the NCIT+Surgery and CRT + IT groups without bulky N2; (C) OS between the NCIT+Surgery and NCIT+CRT groups without bulky N2; (D) OS between the NCIT+Surgery and CRT + IT groups without bulky N2; (E) PFS between the NCIT+Surgery and NCIT+CRT groups with bulky N2; (F) PFS between the NCIT+Surgery and CRT + IT groups with bulky N2; (G) OS between the NCIT+Surgery and NCIT+CRT groups with bulky N2; (H) OS between the NCIT+Surgery and CRT + IT groups with bulky N2. Abbreviation: PFS, progression‐free survival; OS, overall survival; NCIT, neoadjuvant chemoimmunotherapy; CRT, chemoradiotherapy; IT, immunotherapy; PSM, propensity score matching.
**FIGURE S3:** PFS and OS across the NCIT+Surgery, NCIT+CRT, and CRT + IT groups, stratified by the PD‐L1 expression level (< 1% or ≥ 1%) after PSM. (A) PFS between the NCIT+Surgery and NCIT+CRT groups with the PD‐L1 expression < 1%; (B) PFS between the NCIT+Surgery and NCIT+CRT groups with the PD‐L1 expression ≥ 1%; (C) OS between the NCIT+Surgery and NCIT+CRT groups with the PD‐L1 expression < 1%; (D) OS between the NCIT+Surgery and NCIT+CRT groups with the PD‐L1 expression ≥ 1%; (E) PFS between the NCIT+Surgery and CRT + IT groups with the PD‐L1 expression < 1%; (F) PFS between the NCIT+Surgery and CRT + IT groups with the PD‐L1 expression ≥ 1%; (G) OS between the NCIT+Surgery and CRT + IT groups with the PD‐L1 expression < 1%; (H) OS between the NCIT+Surgery and CRT + IT groups with the PD‐L1 expression ≥ 1%; (I) PFS between the NCIT+CRT and CRT + IT groups with the PD‐L1 expression < 1%; (J) PFS between the NCIT+CRT and CRT + IT groups with the PD‐L1 expression ≥ 1%; (K) OS between the NCIT+CRT and CRT + IT groups with the PD‐L1 expression < 1%; (L) OS between the NCIT+CRT and CRT + IT groups with the PD‐L1 expression ≥ 1%. Abbreviation: PFS, progression‐free survival; OS, overall survival; NCIT, neoadjuvant chemoimmunotherapy; CRT, chemoradiotherapy; IT, immunotherapy; PD‐L1, programmed cell death ligand 1; PSM, propensity score matching.
**FIGURE S4:** PFS and OS across the NCIT+Surgery, NCIT+CRT, and CRT + IT groups, stratified by the clinical stage (IIIA or IIIB) after PSM. (A) PFS between the NCIT+Surgery and NCIT+CRT groups with stage IIIA; (B) PFS between the NCIT+Surgery and NCIT+CRT groups with stage IIIB; (C) OS between the NCIT+Surgery and NCIT+CRT groups with stage IIIA; (D) OS between the NCIT+Surgery and NCIT+CRT groups with stage IIIB; (E) PFS between the NCIT+Surgery and CRT + IT groups with stage IIIA; (F) PFS between the NCIT+Surgery and CRT + IT groups with stage IIIB; (G) OS between the NCIT+Surgery and CRT + IT groups with stage IIIA; (H) OS between the NCIT+Surgery and CRT + IT groups with stage IIIB; (I) PFS between the NCIT+CRT and CRT + IT groups with stage IIIA; (J) PFS between the NCIT+CRT and CRT + IT groups with stage IIIB; (K) OS between the NCIT+CRT and CRT + IT groups with stage IIIA; (L) OS between the NCIT+CRT and CRT + IT groups with stage IIIB. Abbreviation: PFS, progression‐free survival; OS, overall survival; NCIT, neoadjuvant chemoimmunotherapy; CRT, chemoradiotherapy; IT, immunotherapy; PSM, propensity score matching.
**FIGURE S5:** PFS and OS across the NCIT+Surgery and NCIT+CRT groups, stratified by the radiographic response to NCIT after PSM. (A) PFS between the NCIT+Surgery and NCIT+CRT groups with a radiographic response of CR or PR; (B) PFS between the NCIT+Surgery and NCIT+CRT groups with a radiographic response of SD; (C) OS between the NCIT+Surgery and NCIT+CRT groups with a radiographic response of CR or PR; (D) OS between the NCIT+Surgery and NCIT+CRT groups with a radiographic response of SD. Abbreviation: PFS, progression‐free survival; OS, overall survival; NCIT, neoadjuvant chemoimmunotherapy; CRT, chemoradiotherapy; CR, complete response; PR, partial response; SD, stable disease; PSM, propensity score matching.
**FIGURE S6:** PFS and OS across the NCIT+Surgery and NCIT+CRT groups, stratified by receipt of adjuvant immunotherapy after PSM. (A) PFS between the NCIT+Surgery and NCIT+CRT groups with adjuvant immunotherapy; (B) PFS between the NCIT+Surgery and NCIT+CRT groups without adjuvant immunotherapy; (C) OS between the NCIT+Surgery and NCIT+CRT groups with adjuvant immunotherapy; (D) OS between the NCIT+Surgery and NCIT+CRT groups without adjuvant immunotherapy; (E) PFS between the NCIT+Surgery and CRT + IT groups with adjuvant immunotherapy; (F) OS between the NCIT+Surgery and CRT + IT groups with adjuvant immunotherapy; (G) PFS between the NCIT+CRT and CRT + IT groups with adjuvant immunotherapy; (H) OS between the NCIT+CRT and CRT + IT groups with adjuvant immunotherapy. Abbreviation: PFS, progression‐free survival; OS, overall survival; NCIT, neoadjuvant chemoimmunotherapy; CRT, chemoradiotherapy; IT, immunotherapy; PSM, propensity score matching.
**TABLE S1:** Baseline characteristics of the two centers.
**TABLE S2:** Baseline Characteristics of the NCIT+Surgery and NCIT+CRT Groups After PSM.
**TABLE S3:** Baseline Characteristics of the NCIT+Surgery and CRT + IT Groups After PSM.

## Data Availability

The data that support the findings of this study are available on request from the corresponding author. The data are not publicly available due to privacy or ethical restrictions.
