# Supplementary material for: Exploratory Investigation Into Perioperative Treatment Strategies for Potentially Resectable Stage III–N2 Driver Gene–Negative Non–Small Cell Lung Cancer in the Immunotherapy Era
Source: Cancer Med. 2026 Mar 8;15(3):e71696. doi: 10.1002/cam4.71696 (PMC12967538; doi:10.1002/cam4.71696)
Supplement: Supplementary file 1 — FIGURE S1: PFS and OS across the NCIT+Surgery, NCIT+CRT, and CRT + IT groups, stratified by the number of metastatic mediastinal lymph node stations (< 2 vs. ≥ 2) after PSM. (A) PFS between the NCIT+Surgery and NCIT+CRT groups with < 2 metastatic mediastinal lymph node stations; (B) PFS between the NCIT+Surgery and CRT + IT groups with < 2 metastatic mediastinal lymph node stations; (C) OS between the NCIT+Surgery and NCIT+CRT groups with < 2 metastatic mediastinal lymph node stations; (D) OS between the NCIT+Surgery and CRT + IT groups with < 2 metastatic mediastinal lymph node stations; (E) PFS between the NCIT+Surgery and NCIT+CRT groups with ≥ 2 metastatic mediastinal lymph node stations; (F) PFS between the NCIT+Surgery and CRT + IT groups with ≥ 2 metastatic mediastinal lymph node stations; (G) OS between the NCIT+Surgery and NCIT+CRT groups with ≥ 2 metastatic mediastinal lymph node stations; (H) OS between the NCIT+Surgery and CRT + IT groups with ≥ 2 metastatic mediastinal lymph node stations. Abbreviation: PFS, progression‐free survival; OS, overall survival; NCIT, neoadjuvant chemoimmunotherapy; CRT, concurrent chemoradiotherapy; IT, immunotherapy; PSM, propensity score matching. FIGURE S2: PFS and OS across the NCIT+Surgery, NCIT+CRT, and CRT + IT groups, stratified by bulky N2 status (present or absent) after PSM. (A) PFS between the NCIT+Surgery and NCIT+CRT groups without bulky N2; (B) PFS between the NCIT+Surgery and CRT + IT groups without bulky N2; (C) OS between the NCIT+Surgery and NCIT+CRT groups without bulky N2; (D) OS between the NCIT+Surgery and CRT + IT groups without bulky N2; (E) PFS between the NCIT+Surgery and NCIT+CRT groups with bulky N2; (F) PFS between the NCIT+Surgery and CRT + IT groups with bulky N2; (G) OS between the NCIT+Surgery and NCIT+CRT groups with bulky N2; (H) OS between the NCIT+Surgery and CRT + IT groups with bulky N2. Abbreviation: PFS, progression‐free survival; OS, overall survival; NCIT, neoadjuvant chemoimmunoth [file CAM4-15-e71696-s001.docx]

**
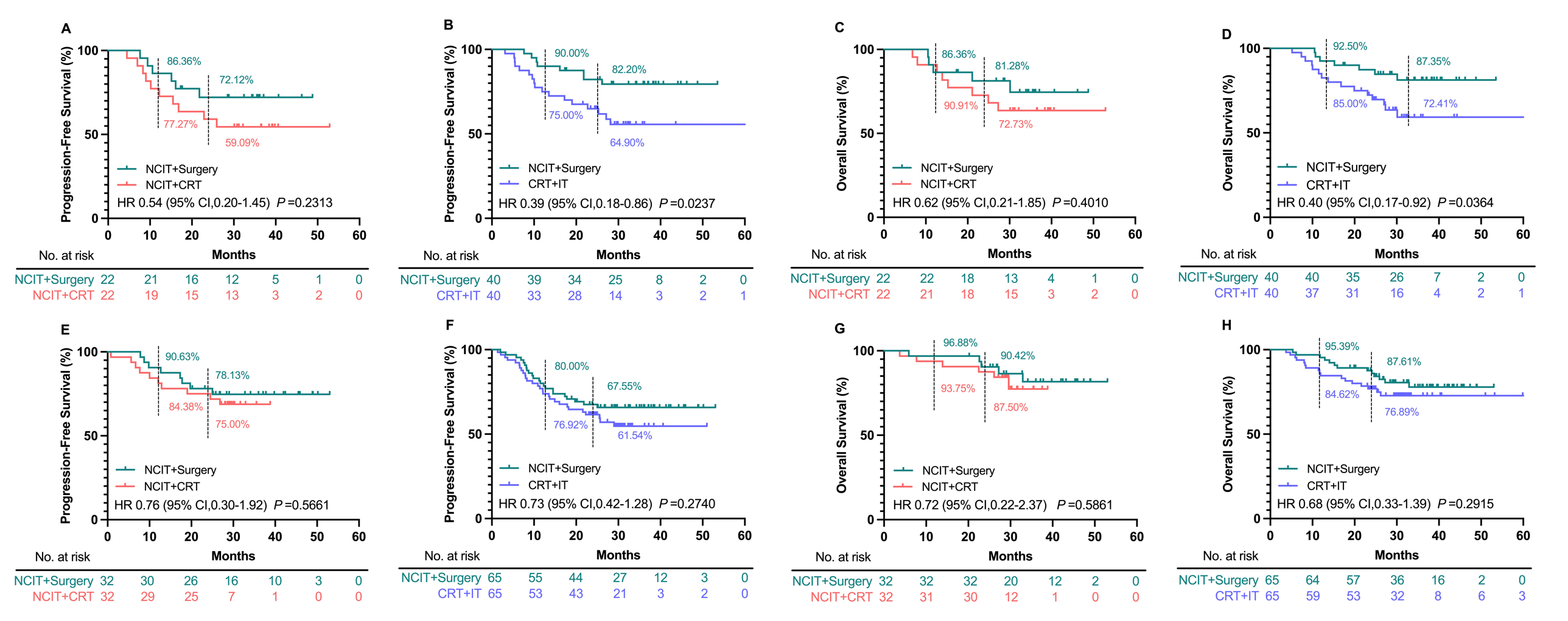
**

**FIGURE S1** PFS and OS across the NCIT+Surgery, NCIT+CRT, and CRT+IT groups, stratified by the number of metastatic mediastinal lymph node stations (< 2 vs. ≥ 2) after PSM. (A) PFS between the NCIT+Surgery and NCIT+CRT groups with < 2 metastatic mediastinal lymph node stations; (B) PFS between the NCIT+Surgery and CRT+IT groups with < 2 metastatic mediastinal lymph node stations; (C) OS between the NCIT+Surgery and NCIT+CRT groups with < 2 metastatic mediastinal lymph node stations; (D) OS between the NCIT+Surgery and CRT+IT groups with < 2 metastatic mediastinal lymph node stations; (E) PFS between the NCIT+Surgery and NCIT+CRT groups with ≥ 2 metastatic mediastinal lymph node stations; (F) PFS between the NCIT+Surgery and CRT+IT groups with ≥ 2 metastatic mediastinal lymph node stations; (G) OS between the NCIT+Surgery and NCIT+CRT groups with ≥ 2 metastatic mediastinal lymph node stations; (H) OS between the NCIT+Surgery and CRT+IT groups with ≥ 2 metastatic mediastinal lymph node stations. Abbreviation: PFS, progression-free survival; OS, overall survival; NCIT, neoadjuvant chemoimmunotherapy; CRT, concurrent chemoradiotherapy; IT, immunotherapy; PSM, propensity score matching.


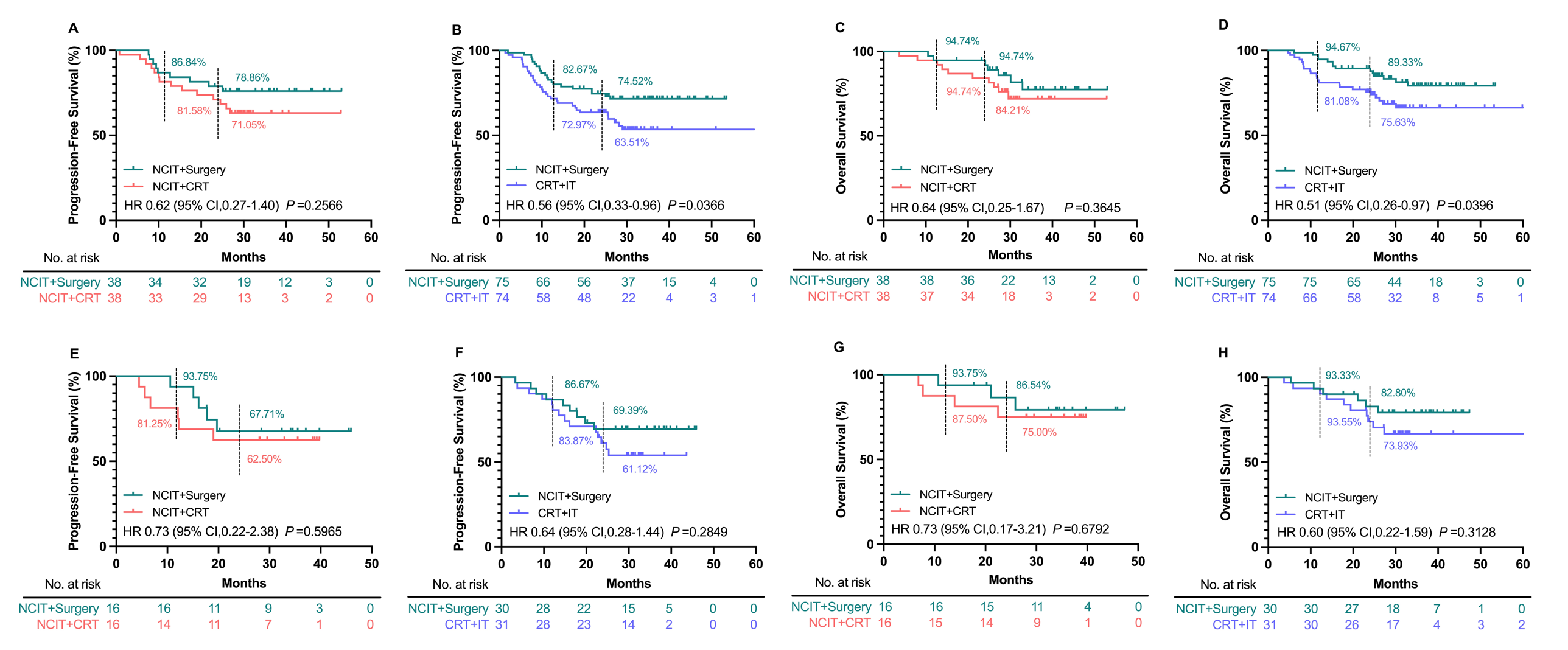


**FIGURE S2** PFS and OS across the NCIT+Surgery, NCIT+CRT, and CRT+IT groups, stratified by bulky N2 status (present or absent) after PSM. (A) PFS between the NCIT+Surgery and NCIT+CRT groups without bulky N2; (B) PFS between the NCIT+Surgery and CRT+IT groups without bulky N2; (C) OS between the NCIT+Surgery and NCIT+CRT groups without bulky N2; (D) OS between the NCIT+Surgery and CRT+IT groups without bulky N2; (E) PFS between the NCIT+Surgery and NCIT+CRT groups with bulky N2; (F) PFS between the NCIT+Surgery and CRT+IT groups with bulky N2; (G) OS between the NCIT+Surgery and NCIT+CRT groups with bulky N2; (H) OS between the NCIT+Surgery and CRT+IT groups with bulky N2. Abbreviation: PFS, progression-free survival; OS, overall survival; NCIT, neoadjuvant chemoimmunotherapy; CRT, chemoradiotherapy; IT, immunotherapy; PSM, propensity score matching.





**FIGURE S3** PFS and OS across the NCIT+Surgery, NCIT+CRT, and CRT+IT groups, stratified by the PD-L1 expression level (< 1% or ≥ 1%) after PSM. (A) PFS between the NCIT+Surgery and NCIT+CRT groups with the PD-L1 expression < 1%; (B) PFS between the NCIT+Surgery and NCIT+CRT groups with the PD-L1 expression ≥ 1%; (C) OS between the NCIT+Surgery and NCIT+CRT groups with the PD-L1 expression < 1%; (D) OS between the NCIT+Surgery and NCIT+CRT groups with the PD-L1 expression ≥ 1%; (E) PFS between the NCIT+Surgery and CRT+IT groups with the PD-L1 expression < 1%; (F) PFS between the NCIT+Surgery and CRT+IT groups with the PD-L1 expression ≥ 1%; (G) OS between the NCIT+Surgery and CRT+IT groups with the PD-L1 expression < 1%; (H) OS between the NCIT+Surgery and CRT+IT groups with the PD-L1 expression ≥ 1%; (I) PFS between the NCIT+CRT and CRT+IT groups with the PD-L1 expression < 1%; (J) PFS between the NCIT+CRT and CRT+IT groups with the PD-L1 expression ≥ 1%; (K) OS between the NCIT+CRT and CRT+IT groups with the PD-L1 expression < 1%; (L) OS between the NCIT+CRT and CRT+IT groups with the PD-L1 expression ≥ 1%. Abbreviation: PFS, progression-free survival; OS, overall survival; NCIT, neoadjuvant chemoimmunotherapy; CRT, chemoradiotherapy; IT, immunotherapy; PD-L1, programmed cell death ligand 1; PSM, propensity score matching.

**

**

**FIGURE S4** PFS and OS across the NCIT+Surgery, NCIT+CRT, and CRT+IT groups, stratified by the clinical stage (IIIA or IIIB) after PSM. (A) PFS between the NCIT+Surgery and NCIT+CRT groups with stage IIIA; (B) PFS between the NCIT+Surgery and NCIT+CRT groups with stage IIIB; (C) OS between the NCIT+Surgery and NCIT+CRT groups with stage IIIA; (D) OS between the NCIT+Surgery and NCIT+CRT groups with stage IIIB; (E) PFS between the NCIT+Surgery and CRT+IT groups with stage IIIA; (F) PFS between the NCIT+Surgery and CRT+IT groups with stage IIIB; (G) OS between the NCIT+Surgery and CRT+IT groups with stage IIIA; (H) OS between the NCIT+Surgery and CRT+IT groups with stage IIIB; (I) PFS between the NCIT+CRT and CRT+IT groups with stage IIIA; (J) PFS between the NCIT+CRT and CRT+IT groups with stage IIIB; (K) OS between the NCIT+CRT and CRT+IT groups with stage IIIA; (L) OS between the NCIT+CRT and CRT+IT groups with stage IIIB. Abbreviation: PFS, progression-free survival; OS, overall survival; NCIT, neoadjuvant chemoimmunotherapy; CRT, chemoradiotherapy; IT, immunotherapy; PSM, propensity score matching.

**
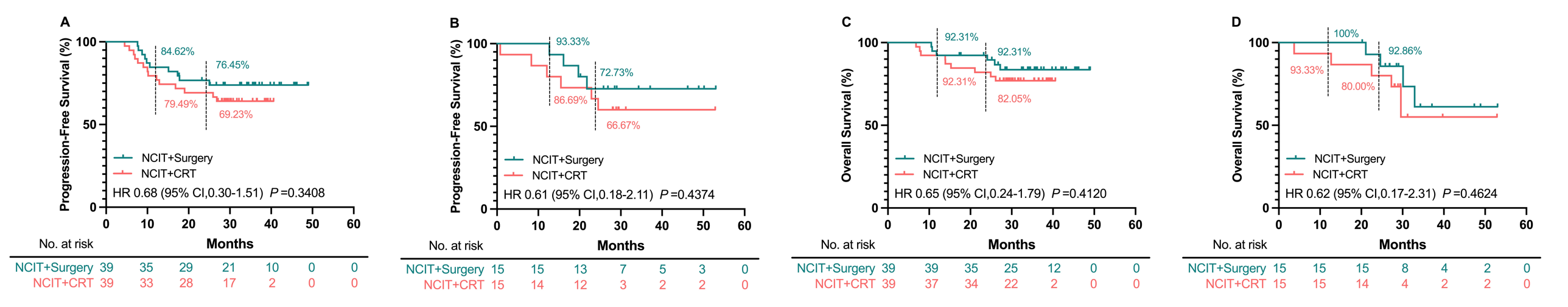
**

**FIGURE S5** PFS and OS across the NCIT+Surgery and NCIT+CRT groups, stratified by the radiographic response to NCIT after PSM. (A) PFS between the NCIT+Surgery and NCIT+CRT groups with a radiographic response of CR or PR; (B) PFS between the NCIT+Surgery and NCIT+CRT groups with a radiographic response of SD; (C) OS between the NCIT+Surgery and NCIT+CRT groups with a radiographic response of CR or PR; (D) OS between the NCIT+Surgery and NCIT+CRT groups with a radiographic response of SD. Abbreviation: PFS, progression-free survival; OS, overall survival; NCIT, neoadjuvant chemoimmunotherapy; CRT, chemoradiotherapy; CR, complete response; PR, partial response; SD, stable disease; PSM, propensity score matching.


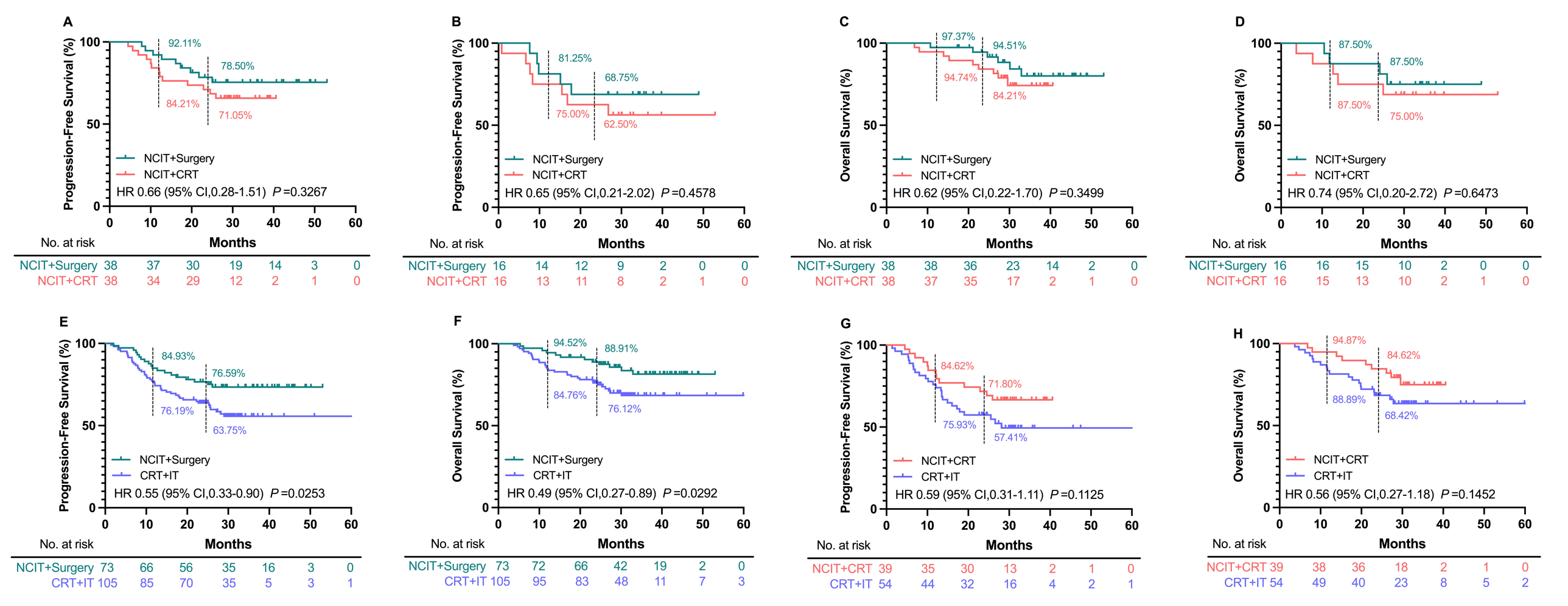


**FIGURE S6** PFS and OS across the NCIT+Surgery and NCIT+CRT groups, stratified by receipt of adjuvant immunotherapy after PSM. (A) PFS between the NCIT+Surgery and NCIT+CRT groups with adjuvant immunotherapy; (B) PFS between the NCIT+Surgery and NCIT+CRT groups without adjuvant immunotherapy; (C) OS between the NCIT+Surgery and NCIT+CRT groups with adjuvant immunotherapy; (D) OS between the NCIT+Surgery and NCIT+CRT groups without adjuvant immunotherapy; (E) PFS between the NCIT+Surgery and CRT+IT groups with adjuvant immunotherapy; (F) OS between the NCIT+Surgery and CRT+IT groups with adjuvant immunotherapy; (G) PFS between the NCIT+CRT and CRT+IT groups with adjuvant immunotherapy; (H) OS between the NCIT+CRT and CRT+IT groups with adjuvant immunotherapy. Abbreviation: PFS, progression-free survival; OS, overall survival; NCIT, neoadjuvant chemoimmunotherapy; CRT, chemoradiotherapy; IT, immunotherapy; PSM, propensity score matching.

**TABLE S1** Baseline characteristics of the two centers.

|  | **Shandong Cancer Hospital**  **(n = 281)** | **Qilu Hospital of Shandong University**  **(n = 82)** | ***P* Value** |
| --- | --- | --- | --- |
| **Age (years)** | 65 (58-69) | 64 (60-68) | 0.672 |
| **Gender** |  |  | 0.743 |
| Male | 250 (89.0%) | 74 (90.2%) |  |
| Female | 31 (11.0%) | 8 (9.8%) |  |
| **Stage** |  |  | 0.526 |
| IIIA | 109 (38.8%) | 35 (42.7%) |  |
| IIIB | 172 (61.2%) | 47 (57.3%) |  |
| **Smoking history** |  |  | 0.813 |
| No | 65 (23.1%) | 20 (24.4%) |  |
| Yes | 216 (76.9%) | 62 (75.6%) |  |
| **Histology** |  |  | 0.758 |
| Squamous | 207 (73.7%) | 59 (72.0%) |  |
| Non-squamous | 74 (26.3%) | 23 (28.0%) |  |
| **Metastatic N2 lymph**  **node status** |  |  | 0.552 |
| N2a | 120 (42.7%) | 32 (39.0%) |  |
| N2b | 161 (57.3%) | 50 (61.0%) |  |
| **Bulky N2 disease** |  |  | 0.891 |
| No | 200 (71.2%) | 59 (72.0%) |  |
| Yes | 81 (28.8%) | 23 (28.0%) |  |
| **PD-L1 expression level** |  |  | 0.733 |
| < 1% | 111 (39.5%) | 29 (35.4%) |  |
| ≥ 1% | 137 (48.8%) | 44 (53.6%) |  |
| Unknown | 33 (11.7%) | 9 (11.0%) |  |
| **ECOG performance status** |  |  | 0.903 |
| 0 | 93 (33.1%) | 25 (30.5%) |  |
| 1 | 185 (65.8%) | 56 (68.3%) |  |
| 2 | 3 (1.1%) | 1 (1.2%) |  |

Abbreviation: PD-L1, programmed death ligand 1; ECOG, eastern cooperative oncology group.

**TABLE S2** Baseline Characteristics of the NCIT+Surgery and NCIT+CRT Groups After PSM.

|  | **NCIT+Surgery**  **(n = 54)** | **NCIT+CRT (n = 54)** | ***P* Value** |
| --- | --- | --- | --- |
| **Age (years)** | 65 (56-69) | 65 (60-70) | 0.579 |
| **Gender** |  |  | < 0.999 |
| Male | 48 (88.9%) | 48 (88.9%) |  |
| Female | 6 (11.1%) | 6 (11.1%) |  |
| **Stage** |  |  | 0.837 |
| IIIA | 17 (31.5%) | 18 (33.3%) |  |
| IIIB | 37 (68.5%) | 36 (66.7%) |  |
| **Histology** |  |  | 0.828 |
| Squamous | 40 (74.1%) | 39 (72.2%) |  |
| Non-squamous | 14 (25.9%) | 15 (27.8%) |  |
| **PD-L1 expression level** |  |  | 0.829 |
| < 1% | 20 (37.0%) | 21 (38.9%) |  |
| ≥ 1% | 27 (50.0%) | 28 (51.9%) |  |
| Unknown | 7 (13.0%) | 5 (9.2%) |  |
| **Radiographic response to NCIT (according to RECIST criteria)** |  |  | < 0.999 |
| CR or PR | 39 (72.2%) | 39 (72.2%) |  |
| SD | 15 (27.8%) | 15 (27.8%) |  |
| **ECOG performance status** |  |  | 0.546 |
| 0 | 21 (38.9%) | 17 (31.5%) |  |
| 1 | 33 (61.1%) | 37 (68.5%) |  |

Abbreviation: NCIT, neoadjuvant chemoimmunotherapy; CRT, chemoradiotherapy; PSM, propensity score matching; PD-L1, programmed death ligand 1; RECIST, response evaluation criteria in solid tumors, CR, complete response; PR, partial response; SD, stable disease; ECOG, eastern cooperative oncology group.

**TABLE S3.** Baseline Characteristics of the NCIT+Surgery and CRT+IT Groups After PSM.

|  | **NCIT+Surgery**  **(n = 105)** | **CRT+IT (n = 105)** | ***P* Value** |
| --- | --- | --- | --- |
| **Age(years)** | 65 (58-69) | 65 (60-70) | 0.670 |
| **Gender** |  |  | 0.622 |
| Male | 97 (92.4%) | 95 (90.5%) |  |
| Female | 8 (7.6%) | 10 (9.5%) |  |
| **Stage** |  |  | < 0.999 |
| IIIA | 35 (33.3%) | 35 (33.3%) |  |
| IIIB | 70 (66.7%) | 70 (66.7%) |  |
| **Histology** |  |  | 0.508 |
| Squamous | 84 (80.0%) | 79 (75.2%) |  |
| Non-squamous | 21 (20.0%) | 26 (24.8%) |  |
| **PD-L1 expression level** |  |  | 0.800 |
| < 1% | 41 (39.0%) | 41 (39.0%) |  |
| ≥ 1% | 50 (47.6%) | 53 (50.5%) |  |
| Unknown | 14 (13.4%) | 11 (10.5%) |  |
| **ECOG performance status** |  |  | 0.599 |
| 0 | 33 (31.4%) | 39 (37.1%) |  |
| 1 | 70 (66.7%) | 65 (61.9%) |  |
| 2 | 2 (1.9%) | 1 (1.0%) |  |

Abbreviation: NCIT, neoadjuvant chemoimmunotherapy; CRT, chemoradiotherapy; IT, immunotherapy; PD-L1, programmed death ligand 1; ECOG, eastern cooperative oncology group.
